# Phenotypic and genotypic identification of hard ticks of the genus *Haemaphysalis* (Acari: Ixodidae) in Peninsular Malaysia

**DOI:** 10.1007/s10493-017-0120-3

**Published:** 2017-04-13

**Authors:** F. C. L. Ernieenor, G. Ernna, A. Mariana

**Affiliations:** Acarology Unit, Infectious Diseases Research Centre, Institute for Medical Research, Jalan Pahang, 50588 Kuala Lumpur, Malaysia

**Keywords:** Ticks, *Haemaphysalis*, *COI* gene, Morphology

## Abstract

Morphotaxonomy based on phenotypic traits of immature hard ticks (Acari: Ixodidae) is a skill challenge and has prompted many inexperienced acarologists to adopt DNA-based methods for identifying and discriminating the species. The aim of this study is therefore to utilize *COI* gene for verifying the morphological status of *Haemaphysalis* ticks in Peninsular Malaysia. A total of 19 on-host ticks collected from four localities were first identified using specific illustrated taxonomic keys that lead to the genus of *Haemaphysalis*. Genotypic traits of tick species were then verified molecularly based on cytochrome oxidase subunit I (*COI*) gene using polymerase chain reaction and direct sequencing. Clustering analysis was carried out by constructing a phylogenetic tree to determine the genetic variation and diversity of local *Haemaphysalis* ticks. Based on external morphological characterizations, all immature ticks were successfully identified down to the genus level only. Molecular analysis of the genotypic using *COI* gene revealed 16 individuals (84%) as *Haemaphysalis hystricis*, and three individuals as *H. humerosa* with sequence homology of 97–99 and 86–87%, respectively. *Haemaphysalis hystricis* were clustered in their respective monophyletic group in the phylogeny trees with a bootstrap of 100%. Furthermore, a low intraspecific variation (<0.3%) was observed among Malaysian *H. hystricis* but high interspecific value (>15%) recorded. This study morphologically and molecularly confirms the presence of *H. hystricis* in Malaysia and the findings will add value to the existing knowledge in identification of ticks in this country.

## Introduction

In tropical countries, ticks are second important arthropods after mosquitoes that have potential to be vectors for transmission of infectious agents including bacteria, viruses and protozoan parasites. Twelve genera comprising 104 species of ticks are found in Southeast Asia with the recent addition of two new species of *Dermacentor* (Apanaskevich and Apanaskevich 2015). In Malaysia, at least 34 tick species belonging to the genera *Amblyomma*, *Dermacentor*, *Haemaphysalis*, *Ixodes* and *Rhipicephalus* have been documented (Hoogstraal et al. 1969; Mariana et al. 2007; Petney et al. 2007; Kolonin 2009). The most species-rich genus in Asia is *Haemaphysalis* with about 52 species or 31% of the world haemaphysalid fauna (Petney et al. 2007). The genus is distributed globally, though the greatest diversity is found in Southeast Asia (Hoogstraal and Trapido 1966; Kolonin 2009).

*Haemaphysalis hystricis* Supino is a three-host tick with a relatively broad host spectrum including human, domestic dogs, wild boar, pigs, buffalo and tigers (Mahara 1997; Cao et al. 2000; Parola et al. 2003). Its distribution occurs throughout the Australasian, Oriental, subtropical and temperate belt of Eastern Asia including Malaysia (Yamaguti et al. 1971). The species is a putative vector of pathogens such as *Ehrlichia*, *Coxiella*, *Trypanosoma* and *Rickettsia* spp., that may cause spotted fever group (SFG), ehrlichiosis and rickettsiosis (Parola et al. 2003; Ando et al. 2010; Arthan et al. 2015; Khoo et al. 2016). Despite its local abundance in Malaysia, most of the information regarding this tick species were published decades ago and little attention has been given to its medical importance and vectorial role. *Haemaphysalis hystricis* has often been misidentified as *H. bispinosa* Neumann, *H birmaniae* Supino, *H. semermis* Neumann and *H. papuana nadchatrami* Hoogstraal (Hoogstraal et al. 1965). To date, the tick-borne diseases of this region remain poorly characterized, mainly due to the limited expertise and accurate information on tick species found in Southeast Asia.

Although the morphological approach for tick identification based on phenotypic traits is economic and convenient, it requires solid training and experience in morphology and taxonomy. The approach is less applicable for damaged ticks and inaccurate for close-related species due to incomplete existing keys for immature stages (Well and Stevens 2008). Subsequently, it is significant to develop more relevant characterization methods in order to differentiate subspecies and species while at the same time offers reliable and convenient technique. Molecular approach, mainly based on mitochondrial (mt) and ribosomal DNA (rDNA) fragments, has provided a complementary tool for accurate identification of ticks (Rumer et al. 2011; Brahma et al. 2014) and characterization of their pathogens (Cheng et al. 2013). Furthermore, molecular identification can be the only technique when there are no other obvious means to match adults with immature stages (Frezal and Leblois 2008; Khera and Vohra 2013). Molecular data can also estimate genetic variation of specific genes directly from the examined taxa and discriminate the closely-related species (Lv et al. 2014; Kanduma et al. 2016).

According to Amendt et al. (2004), polymerase chain reaction (PCR) amplification of suitable regions of the genome, sequence analysis of the amplicons obtained and alignment of the data with reference sequence at various life stages of specimens are the usual and recommended methods to identify organisms. The cytochrome oxidase subunit I (*COI*) is the most frequently used marker and produced highly standard barcode for identification of almost all animal (Hebert et al. 2003). Due to higher mutation rate, maternal inheritance and haploid nature, the mtDNA encoded *COI* gene has been identified as a species-level marker for phylogenetic and taxonomic studies of arthropods including ticks (Casati et al. 2008; Lv et al. 2014; Ernieenor et al. 2016). Caparole et al. (1995) in their study has proven that mtDNA sequences were useful for unraveling the systematics of *Ixodes* ticks while Cakic et al. (2014) successfully discriminated and characterized the *COI* gene of *I. ricinus* ticks in Serbia.

To date, there is no such study on identification of *Haemaphysalis* ticks using well defined molecular approach in Malaysia. Therefore, this study is the first attempt to utilize *COI* gene for verifying the morphological status of *Haemaphysalis* ticks in Malaysia. The genetic species variations and phylogenetic relationship of local *Haemaphysalis* ticks were further determined using clustering analysis based on *COI* sequences.

## Materials and methods

### Collection of tick and morphological identification

**Fig. 1:**
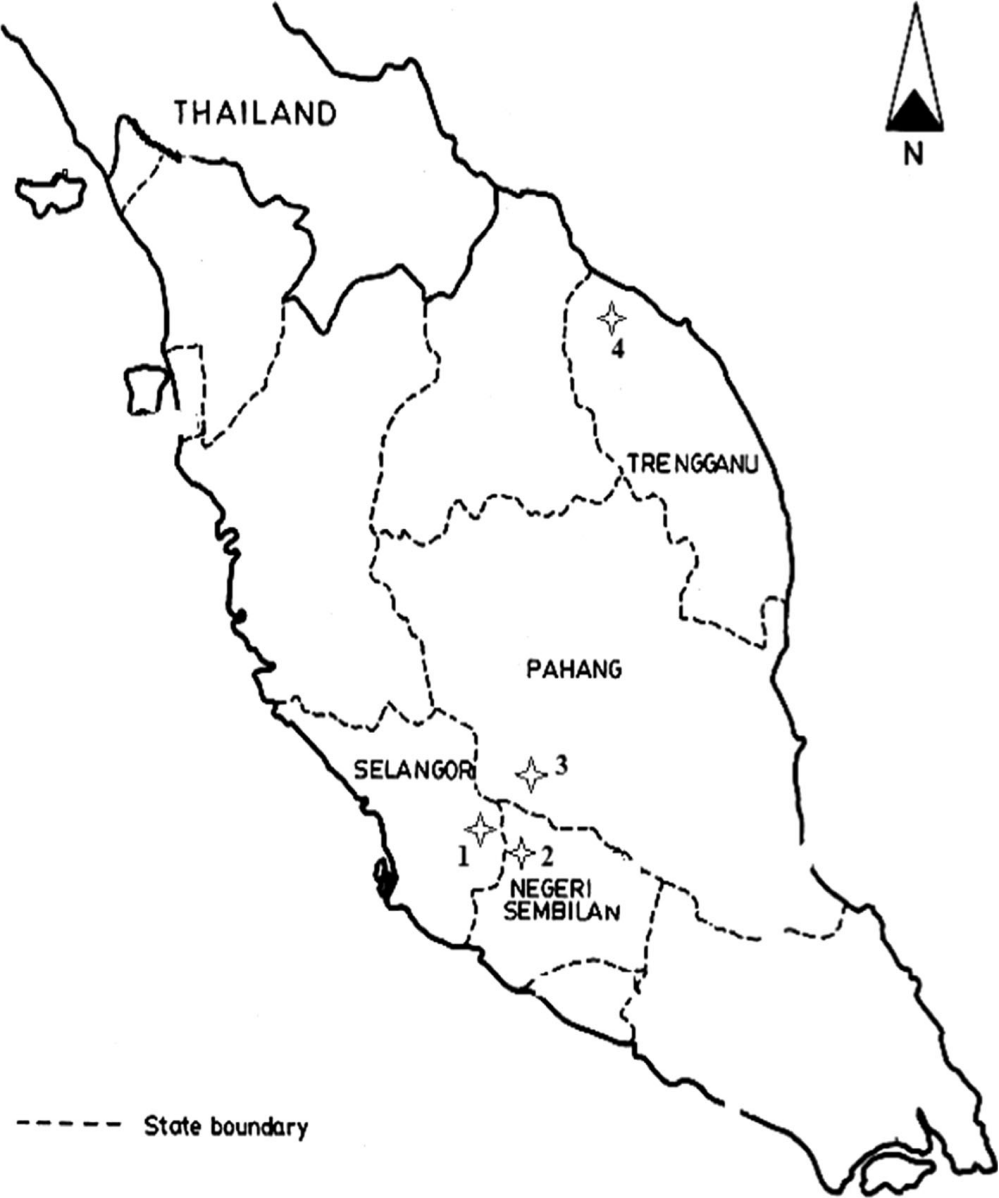
Map of the tick’s collection study sites in Peninsular Malaysia: *1* Hulu Langat, Selangor; *2* Seremban, Negeri Sembilan; *3* Janda Baik, Pahang; *4* Gunung Tebu, Terengganu

Ticks were collected from vertebrate animals caught by live-trapping in four localities (Fig. 1) namely Hulu Langat (Selangor), Janda Baik (Pahang), Seremban (Negeri Sembilan) and Gunung Tebu (Terengganu) between February 2012 and July 2013. Rodent trapping were carried out for four consecutive nights using banana and oil palm fruits as bait. Caught animals were anesthetized with diethyl-ether before screening and the ticks were collected using soft-forceps or sharpened wooden applicators sticks. All experimental procedures involving animals were approved by Animal Use Committee, Ministry of Health Malaysia [Reference Number: ACUC/KKM/02(6)2009] and conducted in accordance to International Conference of Harmonization Good Clinical Practice Guidelines. The ticks were kept individually in vials containing 100% ethanol. All samples were preliminary identified to genus-level based on external morphological characteristics under a stereo microscope (Model Stemi DV4 Zeiss, Germany) using specific illustrated morphological taxonomic keys (Kohls 1957; Walker et al. 2003).

### DNA extraction

DNA extraction was performed using whole body of tick after three times of washing with sterile distilled water. Total genomic DNA was extracted using QIAamp DNA Mini Kit (Qiagen, Germany). The ticks were first macerated using sterile tips for 5 min in 80 μl of sterile phosphate buffer saline (PBS) followed by adding 100 μl of ATL lysis buffer. Samples were then incubated at 56 °C for 6 h after adding 20 μl Proteinase K for complete lyses. The following steps were performed according to manufacturer’s protocol. The DNA was then used for subsequent PCR.

### PCR for the detection of COI gene of ticks

The first PCR was performed using the cycling parameters and primer pairs (cox-1F and cox-1R) from Chitimia et al. (2010). The amplification program consists of a total of 40 cycles: denaturing at 95 °C for 30 s, annealing at 55 °C for 1 min, and extension at 72 °C for 1 min, with an initial denaturation at 95 °C for 5 min. In the primary amplification, the PCR reaction mix of 50 μl which consisted of 25 μl Taq PCR Master Mix 2X, 10 μl of DNA template, 2.5 μl of 0.5 µM of each primer and 10 μl of nuclease free water. Low amplification rates (<50%) were found and this is a common problem in the recovery of *COI* fragment from tick specimen (Lv et al. 2014). To solve this issue, the *COI* of *Haemaphysalis* ticks was amplified using nested PCR in the present study. For the nested PCR amplification, 5 μl of the first amplification product was used as a template with the primers C1-J-1718 and C1-N-2329 (Shao et al. 2001). In the second amplification, the total reaction volume of 50 μl was made up of 25 μl Taq PCR Master Mix 2X, 5 μl of DNA template, 2.5 μl of 0.5 µM of each primer and 15 μl of nuclease free water. The reamplified PCR followed this modified cycling parameters: 94 °C for 5 min, then 35 cycles of 94 °C for 30 s, annealing temperature at 59 °C for 1 min, and extension at 72 °C for 1 min. For each PCR reaction, a negative control containing double distilled water was included. Both PCR reactions were performed using an Eppendorf Master Cycler Personal machine (Eppendorf, Germany). The PCR amplicons were visualized in 1.5% agarose gels electrophoresis stained with SeeNA II Nucleic Acid Stain DNA (Mbiotech, Korea) and viewed under an ultraviolet trans-illuminator.

### DNA sequencing and data analysis

The PCR products was gel-purified using 5 Prime Agarose Gel Extract Mini Kit (Hamburg, Germany) according to manufacturer’s protocols. The purified PCR products were then sent to a commercial sequencing service company, First Base Laboratory Malaysia. Both strands of forward and reverse PCR products were sequenced using the Applied Biosystems BigDye Terminator v3.1 cycle sequencing kit (Applied Biosystem, USA). The obtained sequencing chromatograms were then analyzed and exported as FASTA sequence files. The specimens were molecularly identified by pasting their sequence record in both BLAST (Basic Local Alignment Search Tool) from NCBI’s GenBank and BOLD-IDS tool from BOLD Systems. In GenBank, the nucleotide collection database with MEGABLAST search was used, which is more appropriate for comparing a query to closely related sequences. In BOLD (Barcode of Life Data System), the search was performed with BOLD-IDS tool for animal identification (that use the *COI* barcode) in “Species Level Barcode Records” search database and then in “All Barcode Records on BOLD” search database if the former failed to identify.

The sequences that allowed the species-level identification were aligned with the corresponding sequences of *Haemaphysalis* tick species available in GenBank using CLUSTAL W. Clustering analysis was carried out using Phylogenetic Analysis Using Parsimony (PAUP), version 4.0b10. For distance analysis, a Neighbor-joining (NJ) tree was generated from a Kimura’s two-parameter distance matrix. Maximum parsimony (MP) analysis was performed to determine the most parsimonious tree(s) with a heuristic search of 1000 replications using tree bisection and reconnection option for branch-swapping algorithm. Confidence values for individual branches of the resulting trees were determined through bootstrap analysis with 1000 replicate. The TreeViewX version 0.5.1 software was used to visualize the phylograms obtained from all analyses. In this analysis, *Ixodes granulatus* (GenBank accession no. AB231673) was selected as an outgroup for *COI* gene and one *H. hystricis* sequence (GenBank accession no. JX573137) were aligned simultaneously as a species control.

## Results

**Fig. 2:**
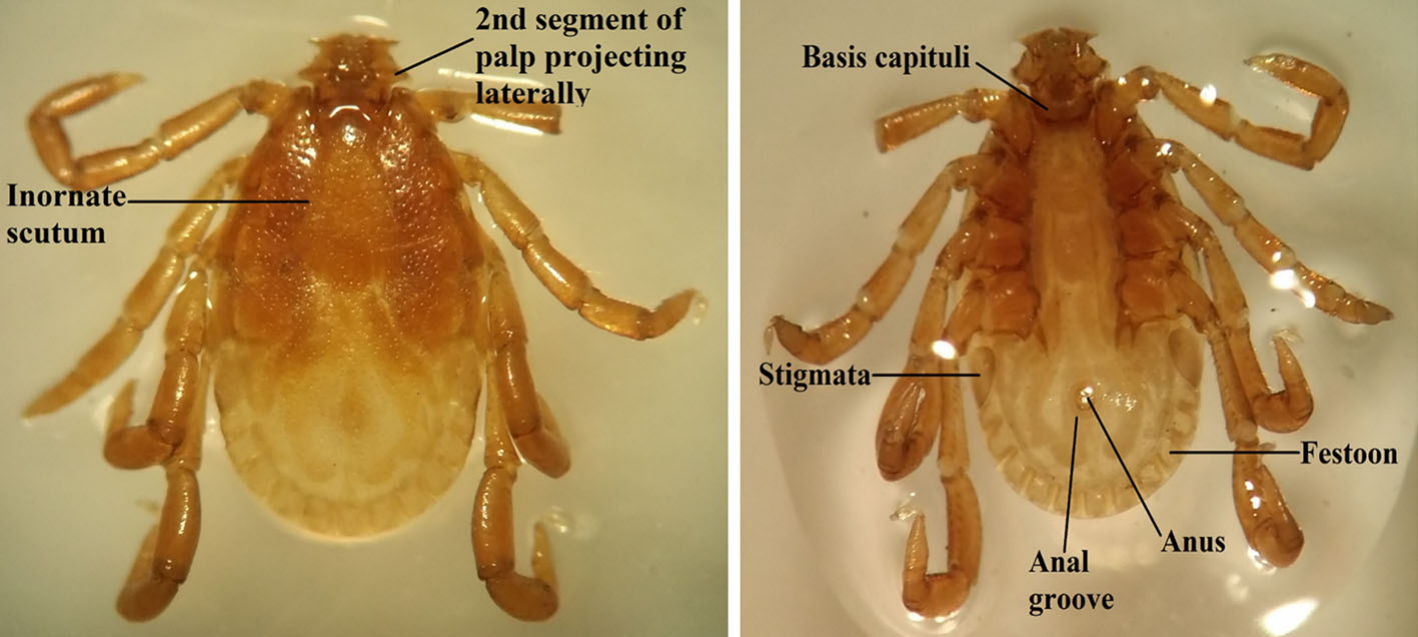
External morphological characteristics of adult male *Haemaphysalis* ticks on dorsal (*left*) and ventral (*right*) view

A total of 19 on-host immature ticks were collected from six species of hosts comprising *Leopoldamys sabanus*, *Sundamys muelleri*, *Rattus tiomanicus*, *Maxomys rajah*, *Rhinosciurus laticaudatus* and *Tupaia glis* (Table 1). The hosts were from the family Muridae, Ptilocercidae and Sciuridae. The ticks collected from all localities were correctly identified as *Haemaphysalis* sp. according to their morphological characters using specific taxonomic keys. Briefly, the unique character of *Haemaphysalis* ticks is their second segment of palps that were laterally produced beyond the basis capituli (Fig. 2). Their eyes are lacking, festoons are present, no ornamentation on scutum and possessed a distinct anal groove embracing the anus posteriorly (Fig. 2). The limitation of this study was that all immature ticks were only identified to the genus level only due to the lack of their morphological descriptions. Those individual immature ticks were therefore subjected to molecular identification.

**Table 1:** Collection of on-host ticks from four localities in Peninsular Malaysia for this study

Locality	Code ID	Species of host	Species ID of tick (morphology)
Hulu Langat, Selangor	HL02_2	*Leopoldamys sabanus*	*Haemaphysalis* sp.
	HL03_1	*Leopoldamys sabanus*	*Haemaphysalis* sp.
	HL07_4	*Leopoldamys sabanus*	*Haemaphysalis* sp.
	HL06_6	*Rhinosciurus laticaudatus*	*Haemaphysalis* sp.
	HL07_15	*Leopoldamys sabanus*	*Haemaphysalis* sp.
	HL03_3	*Leopoldamys sabanus*	*Haemaphysalis* sp.
	HL07_6	*Leopoldamys sabanus*	*Haemaphysalis* sp.
	HL10	*Maxomys rajah*	*Haemaphysalis* sp.
	HL04_18	*Sundamys muelleri*	*Haemaphysalis* sp.
	HL04_13	*Sundamys muelleri*	*Haemaphysalis* sp.
	HL04_10	*Sundamys muelleri*	*Haemaphysalis* sp.
Janda Baik, Pahang	JBB03_1	*Tupaia glis*	*Haemaphysalis* sp.
	JBB03_2	*Tupaia glis*	*Haemaphysalis* sp.
	JBB03_3	*Tupaia glis*	*Haemaphysalis* sp.
Seremban, Negeri Sembilan	SBN26_1	*Rattus tiomanicus*	*Haemaphysalis* sp.
	SBN26_2	*Rattus tiomanicus*	*Haemaphysalis* sp.
Gunung Tebu, Terengganu	GT21_2	*Maxomys rajah*	*Haemaphysalis* sp.
	GT01_13	*Rhinosciurus laticaudatus*	*Haemaphysalis* sp.
	GT01_14	*Rhinosciurus laticaudatus*	*Haemaphysalis* sp.

DNA was extracted from ticks prior to PCR and after partial amplification, the PCR products yielded approximately 630 bp from all samples. Blast analysis of 16 (84%) mitochondrial sequences confirmed the morphological identification of the tick specimens processed by revealing 98–99% and 97.45–99.51% sequence nucleotide similarity with *COI* gene of *Haemaphysalis hystricis* available from GenBank and BOLD, respectively (Table 2). The other three (16%) sequences namely HL06_6, GT01_13 and GT01_14 matched to available sequence of *H. humerosa* with very low nucleotide similarity range from 86 to 87% and 86.39–86.54% for both GenBank and BOLD databases. The mean nucleotide content of the *COI* was 29.4% A, 37.8% T, 18.2% C and 14.5% G. A total of 597 bp fragments were obtained from the multiple alignments of the *COI* gene. Sequence analysis indicated that 144 (24%) variable sites were detected within the *COI* gene and 87 (60%) characters were parsimony informative. Additionally, the conserved sites were constituted by 453 (76%) characters showing that *COI* segment is a very conserved gene in the mtDNA.

**Table 2:** Molecular identification of *Haemaphysalis* ticks (n = 19) from this study compared with those in GenBank and BOLD databases

Code sample	Molecular identification			
	GenBank	Maximum identity (%)	BOLD	Specimen similarity (%)
HL02_2	*H. hystricis*	99	*H. hystricis*	99.51
HL03_1	*H. hystricis*	99	*H. hystricis*	99
Hl07_4	*H. hystricis*	99	*H. hystricis*	99.51
HL06_6	*H. humerosa*	87	*H. humerosa*	86.54
HL07_15	*H. hystricis*	99	*H. hystricis*	99.50
HL03_3	*H. hystricis*	98	*H. hystricis*	98.87
HL07_6	*H. hystricis*	98	*H. hystricis*	98.72
HL10	*H. hystricis*	99	*H. hystricis*	98.87
HL04_18	*H. hystricis*	99	*H. hystricis*	99.35
HL04_13	*H. hystricis*	99	*H. hystricis*	98.38
HL04_10	*H. hystricis*	99	*H. hystricis*	99.34
JBB03_1	*H. hystricis*	99	*H. hystricis*	99.17
JBB03_2	*H. hystricis*	99	*H. hystricis*	97.45
JBB03_3	*H. hystricis*	99	*H. hystricis*	99.19
SBN26_1	*H. hystricis*	99	*H. hystricis*	99.02
SBN26_2	*H. hystricis*	99	*H. hystricis*	98.88
GT21_2	*H. hystricis*	99	*H. hystricis*	98.82
GT01_13	*H. humerosa*	87	*H. humerosa*	86.39
GT01_14	*H. humerosa*	86	*H. humerosa*	86.39

**Fig. 3:**
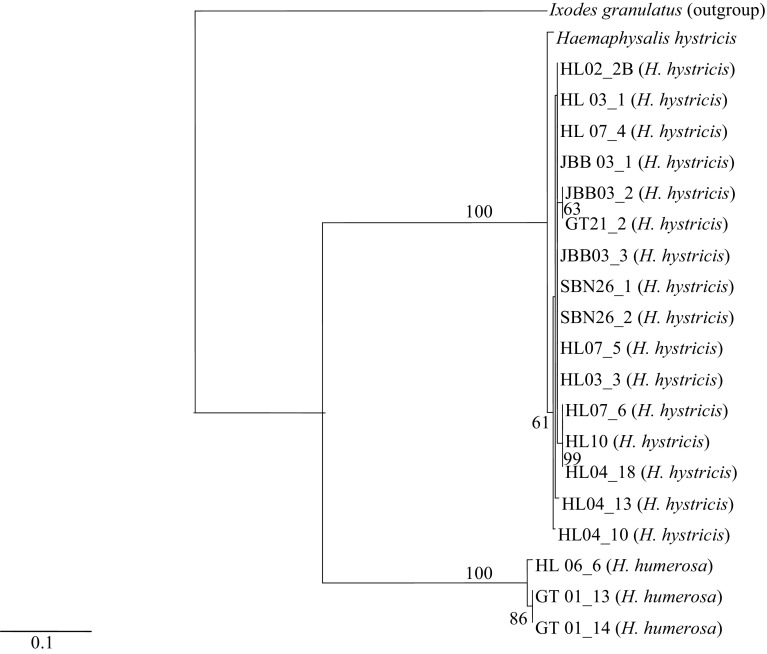
The Neighbor-joining tree generated from 21 sequences (including one outgroup) of *Haemaphysalis hystricis* and *H. humerosa* identified in the present study. The numbers at branches stand for bootstrap values of 1000 replications

**Fig. 4:**
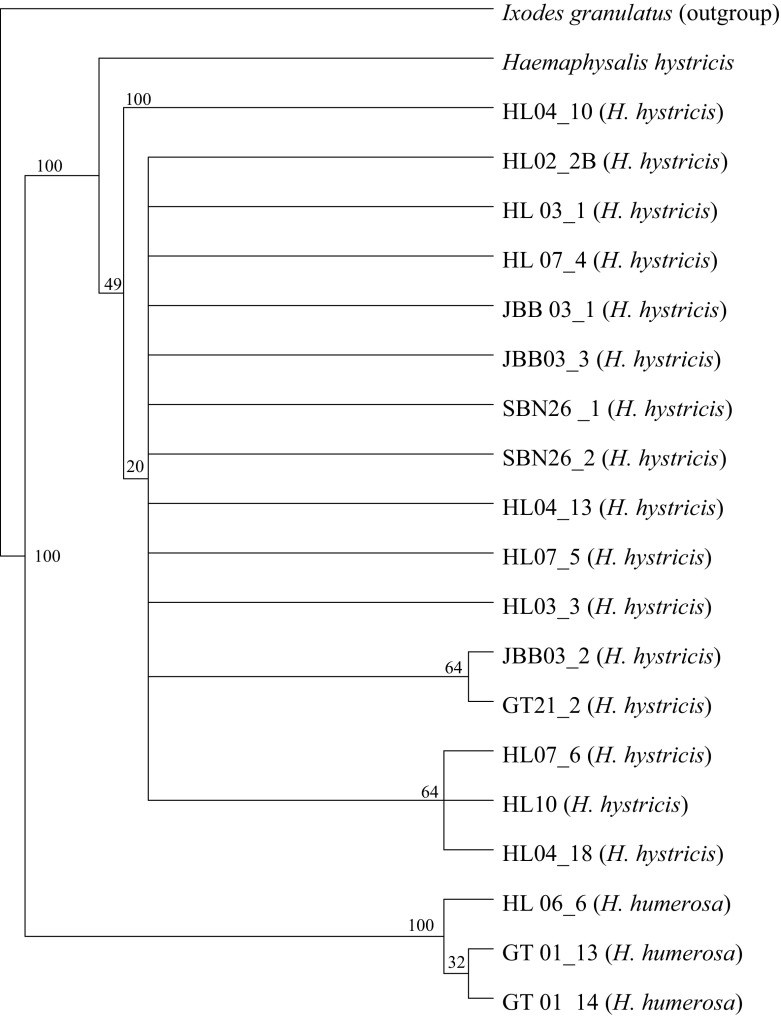
The MP tree generated from 21 sequences (including one outgroup) of *Haemaphysalis hystricis* and *H. humerosa* identified in the present study. The numbers at branches stand for bootstrap values of 1000 replications

Based on clustering analysis, both Neighbor-joining (Fig. 3) and Maximum parsimony (Fig. 4) trees revealed a distinction with high bootstrap value of 100% for *H. hystricis* which can be easily distinguished from other species. Significant grouping of three *H. humerosa* ticks sequences in independent monophyletic subclade was obtained with a bootstrap value of 100% in both analyses. Pairwise distance analysis of *H. hystricis* showed that the local species is genetically different from GenBank species with low genetic distance value ranged from 0.5 to 0.7% (Table 3). Genetic distance analysis of *H. hystricis* collected from all the four localities also indicated a low level of intraspecific value (< 0.3%). However, interspecific distance analyzed by the pairwise comparison revealed that *H. humerosa* sequences genetically differ from *H. hystricis* with high level of genetic variation (15.9–16.1%).

**Table 3:** Genetic distance values of the *COI* DNA sequences (%) amongst the samples

Species	*H. hystricis*	HL 02_2B	HL 03_1	HL 07_4	JBB 03_1	JBB 03_2	JBB 03_3	SBN 26_1	SBN 26_2	HL 04_10	HL 04_13	HL 07_5	GT 21_2	HL 03_3	HL 07_6	HL10	HL 04_18	HL 06_6	GT 01_13	GT 01_14
*H. hystricis*	–																			
HL02_2B	0.5	–																		
HL03_1	0.5	0	–																	
HL07_4	0.5	0	0	–																
JBB03_1	0.5	0	0	0	–															
JBB03_2	0.7	0.2	0.2	0.2	0.2	–														
JBB03_3	0.5	0	0	0	0	0.2	–													
SBN26_1	0.5	0	0	0	0	0.2	0	–												
SBN26_2	0.5	0	0	0	0	0.2	0	0	–											
HL04_10	0.7	0.2	0.2	0.2	0.2	0.3	0.2	0.2	0.2	–										
HL04_13	0.7	0.2	0.2	0.2	0.2	0.3	0.2	0.2	0.2	0.3	–									
HL07_5	0.5	0	0	0	0	0.2	0	0	0	0.2	0.2	–								
GT21_2	0.7	0.2	0.2	0.2	0.2	0	0.2	0.2	0.2	0.3	0.3	0.2	–							
HL03_3	0.5	0	0	0	0	0.2	0	0	0	0.2	0.2	0	0.2	–						
HL07_6	0.7	0.2	0.2	0.2	0.2	0.3	0.2	0.2	0.2	0.3	0.3	0.2	0.3	0.2	–					
HL10	0.7	0.2	0.2	0.2	0.2	0.3	0.2	0.2	0.2	0.3	0.3	0.2	0.3	0.2	0	–				
HL04_18	0.7	0.2	0.2	0.2	0.2	0.3	0.2	0.2	0.2	0.3	0.3	0.2	0.3	0.2	0	0	–			
HL06_6	15.7	15.9	15.9	15.9	15.9	16.1	15.9	15.9	15.9	15.7	15.9	15.9	16.1	15.9	16.1	16.1	16.1	–		
GT01_13	15.7	15.9	15.9	15.9	15.9	16.1	15.9	15.9	15.9	15.7	15.9	15.9	16.1	15.9	16.1	16.1	16.1	0.3	–	
GT01_14	15.7	15.9	15.9	15.9	15.9	16.1	15.9	15.9	15.9	15.7	15.9	15.9	16.1	15.9	16.1	16.1	16.1	0.3	0	–

## Discussion

Some tick genera are associated with various diseases, for example *Haemaphysalis* are disease agents for rickettsial spotted fever, tick typhus, anaplasmosis and ehrlichiosis (Kang et al. 2016; Khoo et al. 2016). Ticks tend to be localized in specific ecosystems but the increased speed and movement of people, ecotourism, translocation of wildlife and climate change provide risks of pathogen spreading beyond their natural ranges (Muruthi et al. 2016). Thus, accurate identification of ticks is of great significance for the investigation of epidemic disease epidemiology and to develop better control measures.

In this study, ticks collected from different localities far apart were confirmed morphologically as the genus *Haemaphysalis*. One of the defining morphological features of this genus is the presence of a prominent “blade-like dorsal retrograde process (Nuttall and Warburton 1915) on trochanter I. They also have short and wide palps with the palp femur projecting laterally beyond the rectangular basis capituli (Hoogstraal and Kim 1985). The dominant hosts of *Haemaphysalis* ticks in our sampling sites were Muridae family comprising of *L. sabanus*, *S. muelleri*, *M. rajah* and *R. tiomanicus*. This observation is consistent with previous studies that reported the abundance of *Haemaphysalis* ticks with 166 valid species (Burger et al. 2013) and their prevalence in domestic animals and rodents surrounding South East Asia (Kolonin 2009). Control of these animals need to be considered if local *Haemaphysalis* ticks were identified as a cause for any potential tick-borne infections.

Findings of the present study have verified the identity of *Haemaphysalis* ticks with high percentage of similarities to *H. hystricis* species as supported not only by the genetic clade but also with those from international databases. Our results clearly indicate the advantages of using *COI* gene that can provide sufficient power in identifying and discriminating species of *Haemaphysalis* ticks. Both NJ and MP tree topology also revealed close grouping of local *H. hystricis* and reference species with 100% bootstrap value. The high bootstrap support of this node may be due to amino acid homoplasy of the *COI* sequences (Burger et al. 2013). Prior to this study, there was only one *COI* sequence for *H. hystricis* that have been published in the GenBank and BOLD database. Therefore, sequences in this study only revealed 99% nucleotide similarity compensating for this lack of consistent data on specimens and a few number of populations from other countries in the databases. Small differences were probably caused by intraspecific variation which explains the polymorphism of this marker (Nava et al. 2010). The present study also revealed the distribution of only one tick species of *H. hystricis* collected from all localities despite the emergence of several known *Haemaphysalis* ticks in Peninsular Malaysia. It is speculated that the widely distribution of this species around Malaysia is probably due to climate and surrounding ecological conditions such as forests, shrub-undergrowth and presence of river at the sampling locality that might favored the survival (Estrada-Pena et al. 2012) of *H. hystricis* ticks.

The low percentage similarity value (86–87%) shown by three samples to corresponding accession sequences of *H. humerosa* species could be associated with the cryptic hybridization factor or geographical separations (Taberlet et al. 1997) which according to Rees et al. (2003) results to nucleotide substitutions. Significant grouping of *H. humerosa* in independent monophyletic clade also showed that small sample size of this species provides little support for intraspecific genetic diversity and phylogenetic inferences (Low et al. 2015). Furthermore, the high dissimilarity value and failure to cluster together with the rest of *H. hystricis* could be attributed to the absence of this species recorded in Malaysia and limited representation of their sequence in GenBank and BOLD. *Haemaphysalis humerosa* ticks have been reported mainly from Australia and can transmit Q fever (Stewart et al. 1987; Hammer et al. 2015).

Regarding to the genetic distance, a low intraspecific variation was observed among *H. hystricis* ticks collected from different localities (0–0.3%), but a high interspecific value (15.9–16.1%) with other species of the same genus. Thus, these observations suggest that mitochondrial *COI* gene is usually informative for determination of genetic variation either by interspecies or intraspecies of ticks. Moreover, tree topologies from different clustering analysis clearly indicated that different geographical in the present study had a smaller source of genetic variation by clustering all *H. hystricis* ticks in one clade. Notably, ecological variables and geographical distance did not explain the local patterns of differentiation observed in *H. hystricis*. This finding is in agreeable with previous studies which reported that short range movement of on-host ticks could explain for the low intraspecific value and similarity of ticks from some localities in Peninsular Malaysia (Fajs et al. 2012; Ernieenor et al. 2016).

The comparison between GenBank and BOLD databases reveals that GenBank had higher success rate in one time BLAST searches. This may probably due to the fact that GenBank presents a most comprehensive, more recent and specific database than BOLD (Benson et al. 2012). Moreover, the distribution of ticks *COI* sequences were more numerous in GenBank and some reference sequences were tagged as barcodes fragment (Sonet et al. 2013) for accurate species identification.

## Conclusion

In conclusion, this study presents phenotypic identification of local *Haemaphysalis* ticks were supported by genotypic analysis using *COI* genetic marker. Our study produced the first *COI* barcoding sequences for *H. hystricis* from different localities in Peninsular Malaysia which contribute to the existing of nucleotide database of ticks. Based on clustering analysis, both NJ and MP tree showed very clear grouping of *H. hystricis* with reference sequences supported by high bootstrap value. Further sampling on a wide geographical of the genus *Haemaphysalis*, particularly *H. hystricis* should be considered to improve our understanding of the taxonomic and genetic variation of this species. The presence of *H. hystricis* species in Malaysia also merits further investigation as a potential vector of tick-borne diseases. The use of *COI* as a standard genetic marker to differentiate and identify tick species in Malaysia is therefore proposed.
